# Sugar tax in Poland: population-level trends in obesity, sugar consumption and healthcare utilization

**DOI:** 10.3389/fpubh.2026.1811509

**Published:** 2026-04-28

**Authors:** Michał Seweryn, Małgorzata M. Bała, Grzegorz Juszczyk, Filip Nowak, Ewa Płaczkiewicz Jankowska

**Affiliations:** 1 Faculty of Health Sciences, Andrzej Frycz Modrzewski University, Krakow, Poland; 2 Epidemiology and Preventive Medicine, Jagiellonian University Medical College, Krakow, Poland; 3 Department of Public Health, Medical University of Warsaw, Warsaw, Poland; 4 National Health Fund, Warsaw, Poland

**Keywords:** health policy, obesity, public health, sin tax, sugar tax

## Abstract

**Objective:**

This ecological study describes population-level trends in obesity, sugar-sweetened beverage (SSB) consumption, per-capita sugar purchases and publicly funded healthcare use for obesity-related conditions in Poland around the introduction of a sugar tax in 2021.

**Methods:**

We analyzed nationally aggregated administrative and survey data from the National Health Fund (NHF), the e-Health Center, the Central Statistical Office (CSO) and the National Public Health Institute (NPHI). Annual time series (2014–2024, where available) on per-capita sugar purchases and healthcare use for obesity and major obesity-related comorbidities were evaluated using segmented time-series regression with a 2021 break-point and a COVID-19 (2020) indicator. Survey-based data on SSB and sweets consumption and on overweight and obesity (2018, 2022, 2025), plus cross-sectional BMI data for 2025, were summarized descriptively.

**Results:**

Per-capita sugar purchases decreased moderately between 2018 and 2024. Survey data indicated an initial reduction in SSB consumption after 2021, followed by partial rebound by 2025, and a shift towards more frequent sweets consumption, while overweight and obesity continued to rise. For obesity-related conditions, numbers of patients receiving disease-specific healthcare showed a COVID-19-related dip in 2020 and subsequent post-2021 increases, with bariatric surgery and pharmacological treatment of obesity also rising over the study period.

**Conclusion:**

In the first 4 years after sugar tax implementation, we observed modest declines in per-capita sugar purchases, transient reductions in SSB consumption and increasing sweets consumption, alongside continued growth in obesity and obesity-related healthcare use. Given the ecological design and short follow-up, these findings represent temporal associations rather than evidence of direct causal effects and underline the need to embed fiscal measures such as a sugar tax within broader, multi-level strategies for obesity prevention, health promotion and access to effective obesity treatment.

## 1 Introduction

Obesity is a complex multifactorial disease defined by the World Health Organization (WHO) as excessive adiposity that presents a risk to health, and it constitutes a major public health concern worldwide. In 2019, an estimated 5 million noncommunicable disease (NCD) deaths were caused by higher-than-optimal BMI (1). The World Obesity Federation’s Atlas 2025 projects that the total number of adults living with obesity will increase by more than 115% between 2010 and 2030, from 524 million to 1.13 billion (2). Such trends, together with 1.6 million premature deaths annually due to NCDs such as diabetes, cancer, heart disease, and stroke associated with overweight and obesity, are alarming and require strong, coordinated public health action (2). Based on the latest Eurostat data for 2022 (published in July 2024), Poland belongs to the more affected countries in the European Union (EU): the overall prevalence of over-weight (BMI ≥ 25 kg/m^**2**^) is above the EU average of 50.6%, with particularly high rates among men (~65%) and substantial rates among women (~55%) (3). Male obesity in Poland (24.2%) is among the highest in the EU, while female obesity (~16.1%) is moderate compared with other Member States (3). Age patterns mirror those in the EU, with the lowest prevalence in young adults (16–24), and the highest in older adults (65–74), Poland’s overall overweight rate is above the EU average of 50.6%, placing it among the more affected countries. Poland’s male obesity rate (24.2%) is among the highest in the EU, just behind Malta. Female obesity in Poland (~16.1%) is moderate—higher than Italy but lower than Latvia and Estonia. Age-related trends in Poland correspond to EU patterns: lowest obesity in youth (16–24), peaking in older adults (65–74), and consistently higher rates of both overweight and obesity in men than in women (3). The gap between male and female obesity is consistent across all age groups, with men showing higher rates of both overweight and obesity (3). WHO European Regional Obesity Report identifies obesity as a significant risk factor and a serious public health challenge globally and a major determinant of disability and death in the WHO European Region (4). Given the unstoppable rise in obesity rates, the WHO has declared obesity one of today’s most important, yet still neglected public health problems (5).

Excess intake of free sugars and high consumption of sugar-sweetened beverages (SSBs) have been linked in numerous studies to increased risks of obesity and multiple di-et-related NCDs, including type 2 diabetes, hypertension, and cardiovascular disease (6–12). These relationships are mediated not only by excess caloric intake, but also by the metabolic and behavioral effects of high-sugar products and ultra-processed foods. Obesity impairs health and quality of life and is a major risk factor for type 2 diabetes, hypertension, atherogenic dyslipidemia, cardiovascular disease, certain cancers, obstructive sleep apnea (OSA), and metabolic dysfunction-associated steatotic liver disease (MASLD), all of which require treatment, strain healthcare systems, and contribute to premature mortality. The primary drivers of the obesity epidemic include excessive consumption of unhealthy processed foods and beverages, which may impair natural hunger–satiety regulation in susceptible individuals. Consumers often have limited understanding of how processed foods and the interaction of biological and psychological factors can lead to chronic overconsumption. Among these drivers, SSBs and highly processed foods, produced and marketed on a large scale, play a leading role (10–12). Reducing population exposure to free sugars and SSBs is therefore a key target for obesity prevention and control.

In response, the WHO recommends limiting free sugars to less than 10% of daily energy intake and endorses taxation of SSBs at a level that increases their retail prices by at least 20% as a public health policy tool (13, 14). Sugar taxes are fiscal instruments designed for two main purposes: (1) to reduce SSB consumption and motivate manufacturers to reformulate products by lowering sugar content, and (2) to generate revenue that can be reinvested in public health activities aimed at reducing sugar intake (health education), preventing diet-related diseases, and supporting their treatment. Experience from Europe illustrates an evolution in the design of these taxes. Early models, such as the tax introduced in France in 2012, were simple volumetric levies (a fixed rate per liter, regardless of sugar content) that had limited impact on product composition because low- and high-sugar beverages were taxed equally (15). A breakthrough was the British Soft Drinks Industry Levy (SDIL), announced in 2016 and implemented in 2018, which introduced a tiered structure based on sugar content with thresholds of 5 g and 8 g per 100 mL (16). This two-tiered levy created strong financial incentives for producers to reformulate their products, reduce portion sizes, and shift consumers toward lower-sugar alternatives (17–19), and its apparent success inspired reforms of existing taxes and adoption of similar models in other countries. For example, France restructured its tax in 2018 toward a progressive model more closely linked to sugar content (15), and Poland, when introducing its own fee in 2021, adopted an advanced hybrid model dependent on sugar content (20). International experience also shows that sugar taxes generate substantial public revenue that can, in principle, be reinvested in healthcare and prevention programs (21), although revenues are inherently unstable and tend to decrease as taxes successfully reduce consumption and encourage reformulation (22).

The construction of the Polish sugar fee is broadly similar to that implemented in Portugal and the United Kingdom, with a base component and an additional charge linked to sugar content (20). A specific feature of the Polish solution is that it also covers sugar-free soft drinks and beverages containing taurine and/or caffeine. The official justification for this extension was to counteract the increasing consumption of energy drinks among adolescents and to prevent the consolidation of unhealthy taste preferences and dietary habits (23). The Government argued that frequent consumption of sweet-tasting beverages, regardless of the caloric value or sweetening agent used, may increase tolerance for sweet taste and appetite, thereby indirectly promoting excess energy intake (23). In Poland, over the first three years of application (2021–2023), the sugar fee generated nearly PLN 4.5 billion in revenues for the National Health Fund (NHF) and the state budget. According to the law, 96.5% of the revenues are to be allocated to the NHF for the prevention and treatment of diseases resulting from inappropriate diet and related to obesity (20). However, a report by the Supreme Audit Office (NIK) published in March 2025 revealed substantial irregularities in the management and accounting of these funds and questioned whether the pro-health objectives of the law had been adequately implemented (24). This raises important questions about how effectively the fiscal potential of the sugar tax has been used to support obesity prevention and care in Poland.

Against this backdrop, there is a need to understand how key health-related indicators have evolved in Poland in the early years following the introduction of the sugar tax. This ecological, population-level time-series study describes national trends in overweight and obesity, per-capita sugar purchases, SSB and sweets consumption, and the utilization of publicly funded healthcare services for obesity and major obesity-related comorbidities in the period 2014–2024, with particular attention to the post-2021 period. The aim is to provide a comprehensive, hypothesis-generating overview of population-level temporal patterns in these indicators in the context of sugar tax implementation, without implying individual-level causal effects.

## 2 Materials and methods

### 2.1 Study design and data sources

This study used an ecological time-series design based on aggregated and publicly available administrative and epidemiological data from national institutions in Poland. In this context, “ecological” means that the unit of analysis is the national population of Poland in each calendar year and that all variables are measured as aggregated, group-level indicators rather than individual-level data. The primary objective of this study was to describe population-level temporal trends in (1) overweight and obesity, (2) sugar purchases and consumption of SSBs and sweets, and (3) utilization of publicly funded healthcare services for obesity and major obesity-related comorbidities in the period 2014–2024, with particular attention to the post-2021 period. The following sources were included:

- National Health Fund (NHF): annual numbers of patients receiving reimbursed bariatric surgery; patients filling prescriptions for obesity medications; and annual numbers of patients receiving care for selected obesity-associated conditions (type 2 diabetes, arterial hypertension, dyslipidemia, knee osteoarthritis requiring endo-prosthesis, sleep apnea, and nonalcoholic fatty liver disease, currently referred to as a metabolic dysfunction-associated steatitis liver disease [MASLD]) for 2014–2024, de-pending on data availability (latest year: 2024).
- Centre for e-Health (CeH): annual prescription data for obesity medications, including the number of patients and dispensed packages, for 2019–2024.
- Central Statistical Office (CSO): per capita sugar consumption (kg per person per year) for 2018–2024.
- National Public Health Institute (NPHI): survey-based data on sugar-sweetened beverage (SSB) and sweets consumption among adults, as reported in the Health Status of the Polish Population series (editions 2018, 2022, and 2025).

A structured overview of all indicators, data sources, variable definitions, aggregation level and analytic approach is provided in Supplementary Table S0.

### 2.2 Data handling and analysis

All datasets had been previously aggregated and anonymized by the data holder; therefore, no ethical approval or informed consent was required. As no individual-level records or identifiers were available, data from different sources could not be linked at the person level. All analyses were therefore conducted at the national population level, and the results are subject to ecological fallacy: associations between changes in SSB consumption, obesity and related outcomes cannot be interpreted as individual-level causal relationships. Descriptive statistics were applied, and temporal patterns were illustrated using tables and figures. Year-to-year percentage changes were calculated to assess trends over time. Where applicable, results specifically refer to reimbursed services (e.g., bariatric procedures financed by NHF) and do not capture private-sector activity.

The selection of indicators was also guided by data availability, continuity over time, and relevance to short- to medium-term changes potentially observable within the available observation window.

### 2.3 Selection of comorbidities

The choice of obesity-related comorbidities included in this analysis — type 2 diabetes, arterial hypertension, dyslipidemia, knee osteoarthritis requiring endoprosthesis, sleep apnea and MASLD — was guided by evidence from the comprehensive review by Bray (25) and the systematic review and meta-analysis by Guh et al. (26). These conditions were prioritized because they are highly prevalent, strongly associated with overweight and obesity, and responsive to changes in body weight over relatively short periods, making them suitable indicators of temporal trends in the context of the 2021 sugar tax. Other obesity-associated conditions, such as certain cancers or asthma, were not included due to their long latency periods and multifactorial etiology, which limit their usefulness for assessing short-term population health changes.

### 2.4 Statistical analysis

Annual time-series data for 2014–2024 (or 2017–2024/2018–2024 depending on the indicator) were analyzed using segmented (interrupted) time-series regression with a breakpoint in 2021, the year of sugar tax implementation. The models included a continuous time variable, an indicator for the post-tax period, a post-tax time trend and a binary indicator for the COVID-19 pandemic year (2020). Segmented time-series regression was applied to annual indicators with sufficient pre- and post-2021 observations (per-capita sugar purchases from CSO and all NHF service-based indicators), whereas survey-based outcomes (frequency of SSB and sweets consumption, prevalence of overweight and obesity, and the 2025 BMI distribution) were analyzed using descriptive statistics only and were not included in the segmented models. Outcomes measured as counts were log-transformed, and regression coefficients were back-transformed and expressed as percentage changes. For chronic cardiometabolic conditions (type 2 diabetes, hypertension, dyslipidemia, sleep apnea, knee osteoarthritis and MASLD), the primary time-series outcomes were the annual numbers of unique adult patients receiving disease-specific healthcare services, rather than the numbers of prescriptions or dispensed packages. We considered service-based indicators more suitable for this analysis, as they are less affected by dosing regimens and pack sizes, avoid double-counting of repeat prescriptions, and are expected to be more sensitive in the short term to changes in diagnosis and healthcare utilization patterns than to long-standing, stable pharmacotherapy use. Where medication-based indicators were available (e.g., patients filling prescriptions for antihypertensive, lipid-lowering or antidiabetic drugs), these were analyzed descriptively to contextualize trends but were not used as primary endpoints in the segmented regression models. For obesity pharmacotherapy, only two pre-tax observations (2019–2020) were available; therefore, a simplified segmented model including a single post-tax indicator and a COVID-19 dummy was used, and results were considered exploratory. Given the ecological, population-level design and the limited number of time points, all segmented time-series models were used to characterize temporal patterns around the introduction of the sugar tax and should be regarded as descriptive and hypothesis-generating, rather than as providing individual-level causal effect estimates.

Segmented time-series regression models and descriptive statistics were performed using Microsoft Excel 365 (Microsoft Corp., Redmond, WA, United States). For survey-based outcomes (e.g., frequency of SSB and sweets consumption, prevalence of overweight and obesity), only descriptive statistics were used. Differences between men and women and changes between survey waves were presented as absolute and relative percentage differences, without formal hypothesis testing, because only aggregated, weighted proportions were available. For the 2025 BMI data, which are available only as a cross-sectional population-level snapshot, we reported descriptive statistics only. These data cover the first 7 months of 2025 and are not directly comparable with previous years; therefore, formal hypothesis testing and pre–post comparisons were not performed.

The interrupted time-series approach applied in this study was used to characterize temporal patterns around the introduction of the sugar tax rather than to estimate causal effects. Given the use of annual aggregated data and the limited number of time points, the models were specified in a simplified form and do not account for potential autocorrelation. Therefore, the results should be interpreted as descriptive and exploratory.

## 3 Results

### 3.1 Shifts in sugar and beverage consumption in the context of the sugar tax

**Figure 1:**
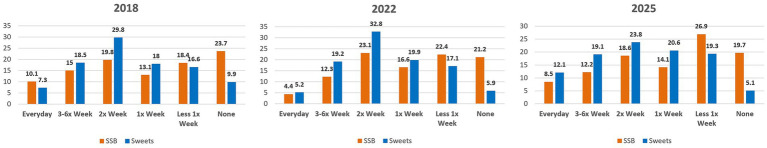
Frequency of consumption of sugar-sweetened beverages (SSBs) and sweets among adults in Poland, overall and by sex, in 2018, 2022, and 2025, based on data from NPHI reports (27–29).

As shown in Figure 1, the frequency of sugar-sweetened beverage (SSB) consumption among adults in Poland shifted substantially between 2018 and 2025, according to the NPHI reports (27–29). Daily consumption declined from 10.1% in 2018 to 4.4% in 2022, possibly reflecting an initial behavioral response to the introduction of the sugar tax in 2021. By 2025, however, the share of daily consumers increased again to 8.5%, suggesting that the early impact may not have been sustained over time.

Moderate consumption patterns fluctuated only slightly over the study period. The proportion of adults drinking SSBs 3–6 times per week decreased from 15.0% in 2018 to 12.3% in 2022 and remained stable in 2025 (12.2%), while twice-weekly consumption peaked in 2022 (23.1%) before falling to 18.6% in 2025. Low-frequency consumption (less than once per week) increased steadily from 18.4% in 2018 to 26.9% in 2025, accompanied by a gradual decline in non-consumers (from 23.7 to 19.7%).

Gender-specific trends mirrored the overall patterns but differed in magnitude. Among men, daily SSBs intake declined from 13.5% in 2018 to 6.8% in 2022, then rose to 10.4% in 2025. Among women, daily consumption was consistently lower (7.1, 2.3, and 6.8%), while abstention was more common (28.9, 25.6, and 22.9% vs. 17.9, 16.3, and 16.1% among men).

These patterns contrast with those observed for sweets consumption, which is not subject to the sugar tax. Daily intake declined modestly from 7.3% in 2018 to 5.2% in 2022, then rose sharply to 12.1% in 2025, surpassing daily SSBs consumption in that year. Occasion-al consumption (less than once per week) also increased slightly (16.6, 17.1, and 19.3%), while moderate consumption (e.g., twice weekly) remained consistently high, peaking at 32.8% in 2022. Gender differences were evident, with women reporting higher daily sweet intake in 2025 (14.0% vs. 10.0% among men), alongside similar distributions in moderate frequency categories.

Market research data from 2024 (30) further supports this interpretation. According to analyses of beverage consumption in Poland, 31.9% of consumers aged 15–75 reported consuming carbonated beverages, while 68.1% did not. Among those who did consume such drinks, 39.2% reported intake no more than twice per month, 30.5% consumed them 1–2 times per week, 14.8% consumed them 3–4 times per week, 5.9% consumed them 5–6 times per week, and 9.7% reported daily use. Importantly, there has been a notable shift toward lower-calorie alternatives: in the last quarter of 2024, 77% of respondents reported drinking regular carbonated beverages, while 60% reported consuming diet or light versions, reflecting growing consumer awareness of sugar content and healthier choices.

Data from Statistics Poland (CSO) (31, 32) complement these findings by providing insights into overall annual sugar intake at the population level. As shown in Table 1, aver-age per-capita sugar consumption decreased from 47.0 to 39.8 kg per person per year be-tween 2018 and 2024. In a segmented time-series analysis with a breakpoint in 2021, the pre-tax period (2018–2020) was characterized by a non-significant downward trend of roughly 10% per year (β_time = −0.11; 95% CI − 0.35 to 0.13; *p* = 0.19). After accounting for this underlying trend and the disruption related to the COVID-19 pandemic year 2020, the introduction of the sugar tax in 2021 was not associated with a statistically significant immediate level change in sugar consumption (β_post = 0.24; 95% CI − 0.39 to 0.86; *p* = 0.25) or with a clear change in the post-tax slope (β_slope post = 0.10; 95% CI − 0.15 to 0.35; *p* = 0.24). The coefficient for the COVID-19 year was likewise non-significant (β_covid2020 = 0.13; 95% CI − 0.28 to 0.54; *p* = 0.31). Overall, within the short observation window available, no distinct break in the trajectory of per-capita sugar consumption following the introduction of the tax could be demonstrated (full regression output in Supplementary Table S1). While the reasons for this decline are not yet clear, it may suggest emerging changes in consumption patterns. Further monitoring will be necessary to determine whether this represents the beginning of a sustained downward trend or a temporary fluctuation.

**Table 1:** Average annual per capita sugar consumption in Poland (kilograms per person), 2018–2024, based on data from Statistics Poland (CSO) (31, 32).

Year	2018	2019	2020	2021	2022	2023	2024
Per capita consumption	47.0	42.1	42.9	41.8	42.8	43.2	39.8

In addition to self-reported consumption and per-capita sugar purchases, external market monitoring data provide some insight into short-term price and volume responses to the tax. According to analyses by the Market Monitoring Centre and the Supreme Audit Office, retail prices of sweetened non-alcoholic beverages increased after the introduction of the sugar tax in 2021 (by approximately 36% for carbonated drinks, 22% for flavored waters and 18% for iced teas), while sales volumes of these categories declined by about 20, 21 and 5%, respectively (24, 33). These indicators are derived from commercial sales data and are reported here to contextualize the population-level trends observed in our data; they were not included in the interrupted time-series models because the under-lying annualized series were not available to the authors.

### 3.2 Evolving trends in overweight and obesity in Poland

**Figure 2:**
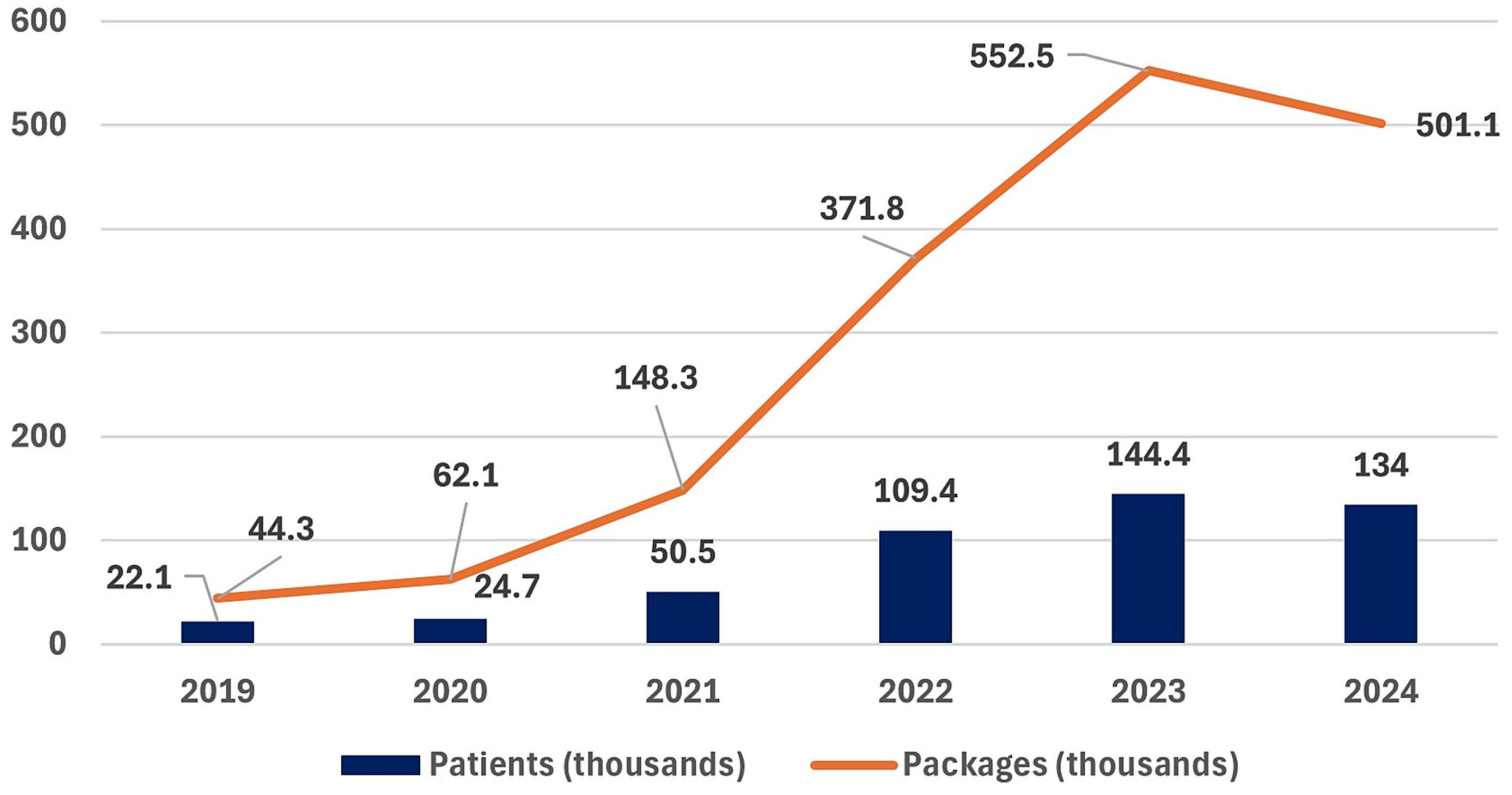
Trends in overweight and obesity in Poland (2018–2025) based on data from NPHI reports (27–29).

Data from NPHI reports (Figure 2) for 2018, 2022, and 2025 (27–29), summarizing their representative population study on health behaviors, reveal concerning upward trends in overweight, obesity, and sugar consumption among the adult population in Poland. Despite the introduction of the sugar tax in 2021, the prevalence of overweight (BMI ≥ 25 kg/m2) continued to rise. In 2018, 58.8% of men and 41.1% of women were overweight. By 2022, these figures had increased to 62 and 43%, respectively, and the 2025 NPHI report indicates further growth to 64% of men and 48% of women.

Obesity (BMI ≥ 30 kg/m2) showed a similar pattern (27–29). In 2018, 11.2% of men and 11.3% of women were obese. By 2022, prevalence increased to 16% among men and 12% among women, and in 2025 it reached 15% in men and 13% in women.

From January 2025, the NHF introduced an obligation for primary care physicians to examine patients for height and weight and report their BMI. Despite the fact that these data do not allow for an interesting comparative analysis from the perspective of our work (before and after the introduction of the sugar tax), we decided to present the latest available data from the period January–July 2025, because in our opinion these are currently the most reliable data on the actual situation regarding overweight and obesity in the Polish population (the survey covered 13.4 million individuals). The data in Table 2 (34) show that 5.05 million individuals (37.7%) were classified as overweight (BMI 25–30), while 3.60 million individuals (26.9%) were classified as obese (BMI ≥ 30), indicating that nearly two-thirds of the adult Polish population are affected by excessive body weight. If subsequent NHF-reported data from primary care confirm these findings, it may turn out that the actual situation regarding obesity in Poland is considerably worse than suggested by previous NPHI survey-based estimates.

**Table 2:** Distribution of the study population by BMI category in Poland, January–July 2025 based on data from NHF (34).

BMI category	BMI range (kg/m^2^)	Number of individuals	Percentage (%)
Underweight	<18.5	233,233	1.74
Normal weight (lower range)	18.5–23	2,359,427	17.60
Normal weight (upper range)	23–25	2,163,056	16.14
Overweight (grade 1)	25–27.5	2,714,566	20.25
Overweight (grade 2)	27.5–30	2,336,589	17.43
Obesity class I (lower range)	30–32.5	1,628,018	12.15
Obesity class I (upper range)	32.5–35	922,271	6.88
Obesity class II	35–40	768,026	5.73
Obesity class III	≥40	277,415	2.07
Total	—	13,402,601	100.00

### 3.3 Healthcare utilization for obesity management

In recent years, bariatric surgery has become a transformative intervention in the treatment of severe obesity. As emphasized in the literature, metabolic and bariatric surgery remains the most effective and durable treatment for severe obesity (35). At the same time, pharmacological therapy is gaining increasing importance in clinical practice and patient care (36).

**Figure 3:**
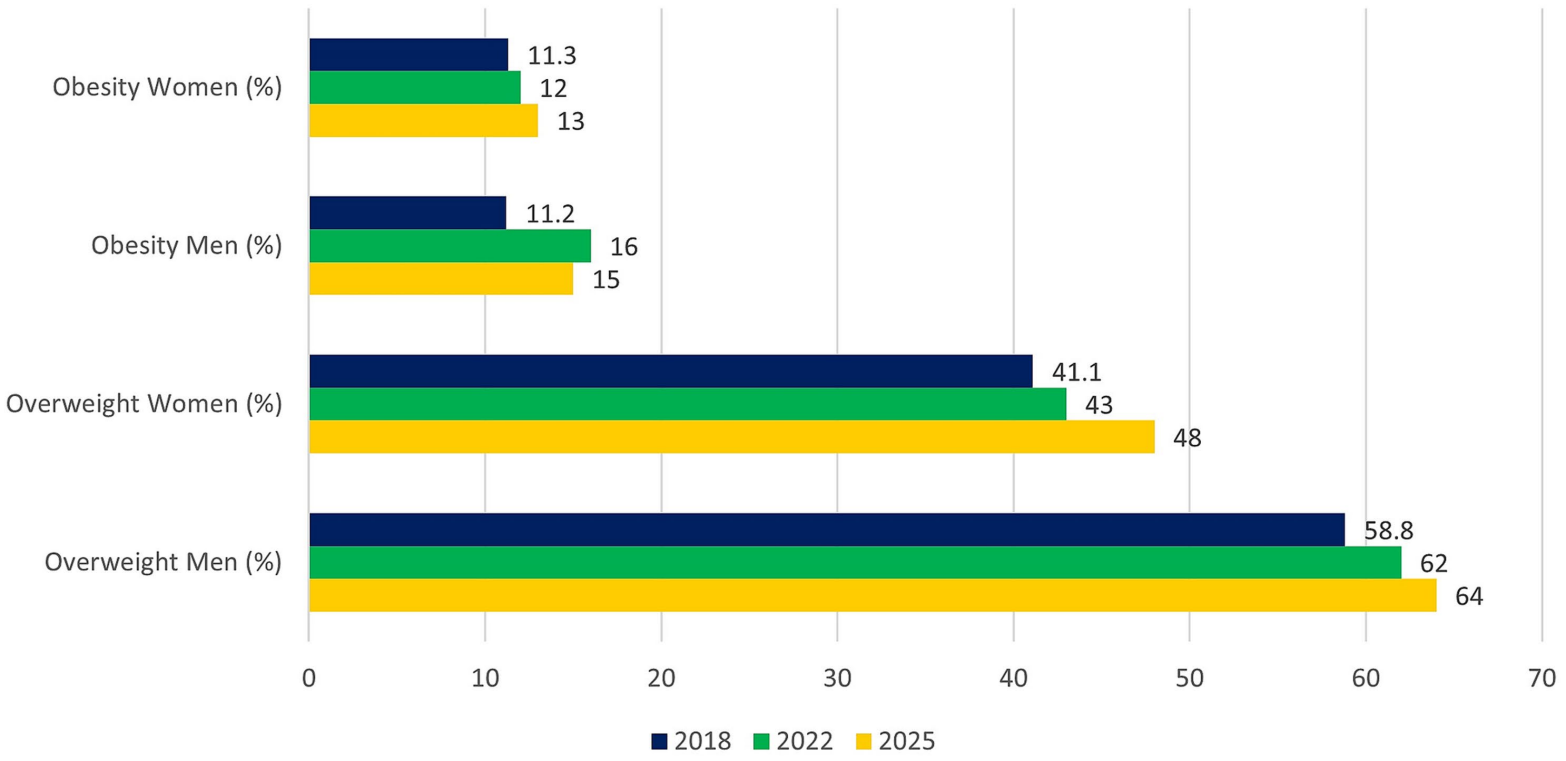
Number of patients undergoing reimbursed bariatric surgery in Poland between 2017 and 2024, based on data from the National Health Fund (NHF) (37). X-axis: year; Y-axis: number of patients.

NHF data [Figure 3; (37)] show that the number of patients undergoing reimbursed bariatric surgery in Poland had risen from 3,300 in 2017 to 7,700 in 2024. In segmented time-series analysis, the pre-tax period was characterized by a steep and statistically significant upward trend of approximately 17% per year (β_time = 0.155; 95% CI 0.089 to 0.221; *p* = 0.0049). The COVID-19 pandemic year 2020 was associated with a marked, around 31% reduction in the number of procedures compared with the underlying trend (β_covid2020 = −0.374; 95% CI − 0.543 to −0.204; *p* = 0.0059). After accounting for both the pre-existing trend and the COVID-19 shock, the introduction of the sugar tax in 2021 was associated with a non-significant immediate level decrease (β_post = −0.187; 95% CI − 0.406 to 0.031; *p* = 0.0717) and no clear change in the post-tax slope (β_slope post = −0.022; 95% CI − 0.100 to 0.055; *p* = 0.43). Overall, these results suggest a continuation of the strong upward trajectory in bariatric surgery utilization, with a temporary pandemic-related dip but no statistically robust evidence of an additional break in level or slope after 2021 (full regression output in Supplementary Table S2).

No medication is currently reimbursed for the treatment of obesity in Poland. Nevertheless, several pharmacological agents, including GLP-1 analogues and other approved drugs, are increasingly prescribed and dispensed.

**Figure 4:**
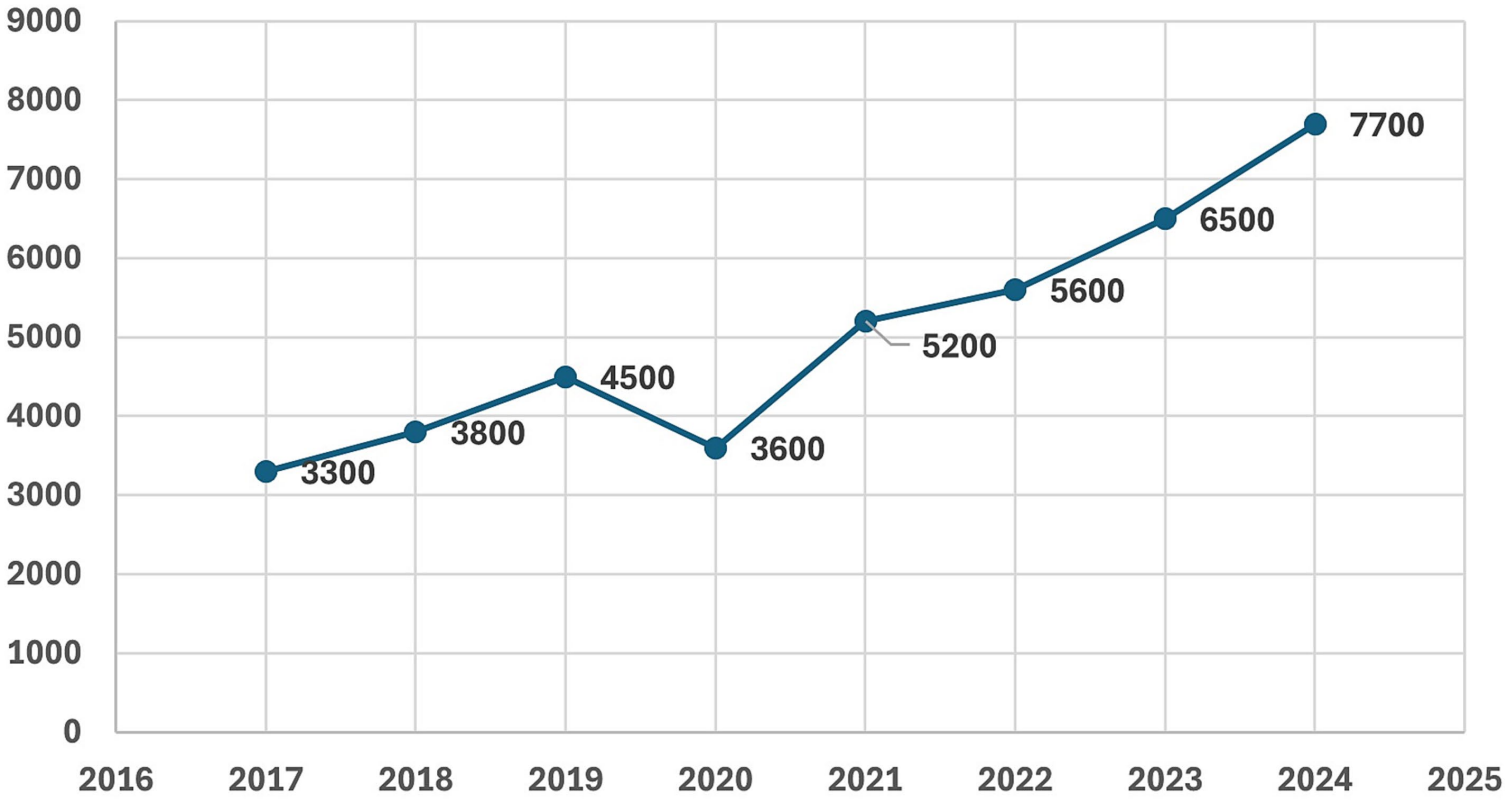
Number of patients (bars, in thousands) and number of packages sold (line, in thousands) for obesity pharmacotherapy in Poland, 2019–2024, based on NHF and CSIOZ data (37). X-axis: year; Y-axis: number of patients/packages (thousands).

As shown in Figure 4, data from the e-Health Center (CSIOZ) (37) indicate a marked and consistent increase in the number of patients receiving pharmacological treatment for obesity in Poland between 2019 and 2024. The number of treated patients rose from ap-proximately 22,000 in 2019 to 144,000 in 2023, followed by a slight decline to 134,000 in 2024, while the number of dispensed medication packages increased from 44,000 in 2019 to 553,000 in 2023 and then decreased to 501,000 in 2024. In a simplified segmented log-linear model including time, a post-2021 indicator and a COVID-19 dummy, we observed a steep positive time trend corresponding to an average increase of approximately 38% per year (β_time = 0.32; 95% CI − 0.26 to 0.90; *p* = 0.14). However, given the very short pre-tax period (two observations), this estimate was imprecise and did not reach conventional statistical significance. The post-2021 indicator (β_post = 0.40; 95% CI − 2.08 to 2.89; *p* = 0.56) and the COVID-19 coefficient (β_covid2020 = −0.21; 95% CI − 2.13 to 1.71; *p* = 0.68) were likewise highly uncertain and non-significant. The modest decline observed in 2024 compared with 2023 should therefore be interpreted with caution and may reflect short-term fluctuations or evolving prescribing patterns rather than a clear reversal of the overall upward trend; additional years of observation will be needed to clarify this pattern. Additionally, this sharp rise, particularly evident from 2021 onwards, likely reflects the growing availability and clinical adoption of modern anti-obesity pharmacotherapy, including GLP-1 receptor agonists (liraglutide at that time) and combination regimens. It should be noted that during this period semaglutide and tirzepatide were not yet available for the treatment of obesity; the semaglutide formulation available in Poland was registered only for type 2 diabetes and was therefore not included in the analyses. We consider this analysis purely exploratory and refrain from attributing changes in obesity pharmacotherapy directly to the sugar tax; however, this observation is noteworthy in the context of the growing rates of overweight and obesity in the population. Full regression results are provided in Supplementary Table S3.

### 3.4 Comorbidities associated with obesity

#### 3.4.1 Type 2 diabetes

Type 2 diabetes is strongly associated with overweight and obesity and accounts for more than 90% of all diabetes cases worldwide (38). In Poland, precise data on the prevalence of type 2 diabetes are not available; however, aggregate data on the overall number of patients with diabetes allow for the analysis of national trends.

**Figure 5:**
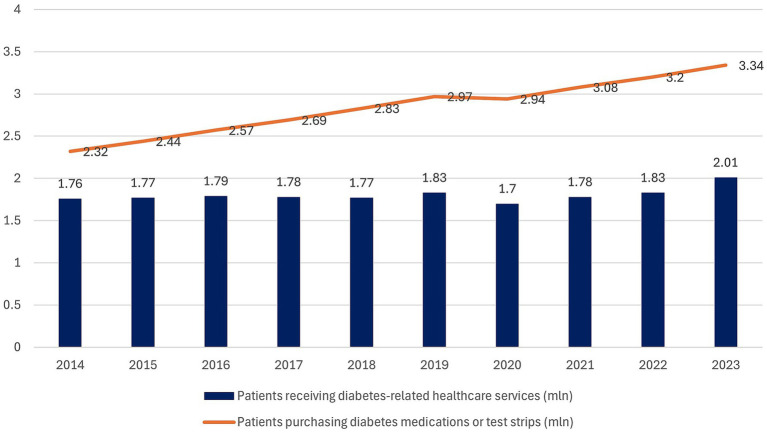
Diabetes-related healthcare service use (bars, in millions) and medication/test strip purchases (line, in millions) in Poland, 2014–2024, based on NHF data (37). X-axis: year; Y-axis: number of patients (millions).

According to NHF data (37), the number of patients receiving diabetes-related healthcare services in Poland (Figure 5) remained relatively stable between 2014 and 2019, ranging from 1.76 to 1.83 million. A decline was observed in 2020 (1.70 million), coinciding with the COVID-19 pandemic, followed by a gradual increase that reached 2.08 million in 2024. In segmented log-linear time-series analysis, the pre-tax period showed no clear underlying trend (β_time = 0.005; 95% CI − 0.003 to 0.014; *p* = 0.17), corresponding to an annual change of roughly +0.5% in the number of patients. After accounting for this background pattern, the year 2021 was not associated with a statistically significant immediate level change (β_post = −0.021; 95% CI − 0.084 to 0.016; *p* = 0.15), whereas the post-2021 slope was significantly steeper, with an additional annual increase of about 5.2% (β_slope post = 0.051; 95% CI 0.033 to 0.069; p ≈ 0.0005). The COVID-19 year 2020 was associated with an approximately 6–7% reduction in service use compared with the underlying trend (β_covid2020 = −0.067; 95% CI − 0.115 to −0.018; p ≈ 0.015).

In contrast, the number of patients purchasing diabetes medications or test strips (at least one purchase per year) demonstrated a steady upward trajectory throughout the entire period, rising from 2.32 million in 2014 to 3.51 million in 2024. This reflects a consistent growth in medication and test strip utilization, outpacing the increase in the number of patients receiving healthcare services.

Overall, these patterns indicate a modest acceleration in the utilization of diabetes-related healthcare services after 2021, alongside a long-term, steady rise in medication and test strip use. Given the observational nature of the data and the presence of other secular and system-level influences (including recovery from the COVID-19 pandemic), these trends should not be interpreted as direct causal effects of the sugar tax. Full regression results for healthcare service use are provided in Supplementary Table S4.

#### 3.4.2 Arterial hypertension

The causal relationship between obesity and hypertension has been consistently demonstrated, with several key mechanisms well described (39). Population-based studies have repeatedly shown strong correlations between body mass index (BMI) and blood pressure, regardless of adiposity level (40, 41).

**Figure 6:**
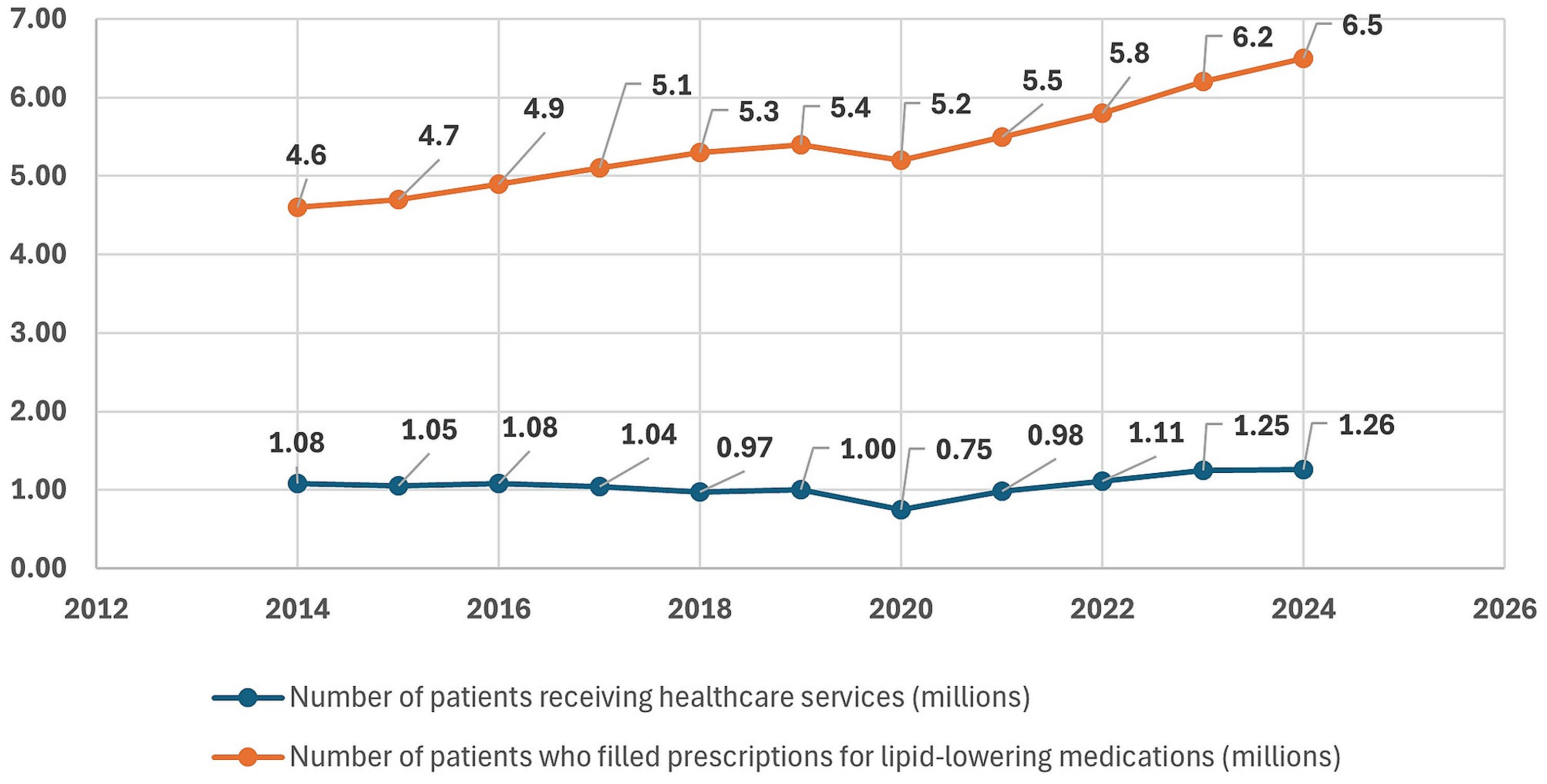
Number of adult patients (millions) receiving healthcare services with a primary diagnosis of hypertension and number of adults who filled prescriptions for reimbursed antihypertensive medications, 2014–2024, based on data from the National Health Fund (NHF) (37). X-axis: year; Y-axis: number of patients (millions).

Figure 6 illustrates the number of adult patients in Poland with a primary diagnosis of hypertension who received healthcare services, compared with the number who filled prescriptions for reimbursed antihypertensive medications between 2014 and 2024. Prescription uptake remained stable at approximately 8.4–9.1 million individuals annually, without visible changes in trend. In contrast, the number of patients receiving healthcare services had been declining steadily from 2014, reaching a low point in 2020 (5.3 million), coinciding with the COVID-19 pandemic. Since 2021, however, this trend has reversed, with a clear upward trajectory emerging and becoming particularly pronounced in 2024, when the number of patients increased to 6.5 million. Overall, these data indicate stable use of antihypertensive medications alongside a recent rebound in healthcare service utilization.

In segmented log-linear time-series analysis, the pre-tax period was characterized by a statistically significant downward trend of about 3.5% per year in the number of patients receiving hypertension-related healthcare services (β_time = −0.036; 95% CI − 0.047 to −0.024; *p* < 0.001). After accounting for this background decline, the sugar-tax year 2021 was not associated with a significant immediate level change (β_post = 0.014; 95% CI − 0.053 to 0.083; *p* = 0.61), whereas the post-2021 slope was markedly steeper, indicating an additional annual increase of roughly 10.7% (β_slope post = 0.101; 95% CI 0.077 to 0.126; *p* < 0.001), consistent with a reversal from a declining to a growing trend (net ≈ +7% per year after 2021). The COVID-19 year 2020 was associated with an estimated 3% reduction compared with the underlying trend (β_covid2020 = −0.031; 95% CI − 0.096 to 0.035; *p* = 0.30), although this effect was not statistically significant. Given the observational nature of the data and the presence of other secular and system-level influences, these changes should not be interpreted as direct causal effects of the sugar tax. Full regression results are provided in Supplementary Table S5.

#### 3.4.3 Dyslipidemia

**Figure 7:**
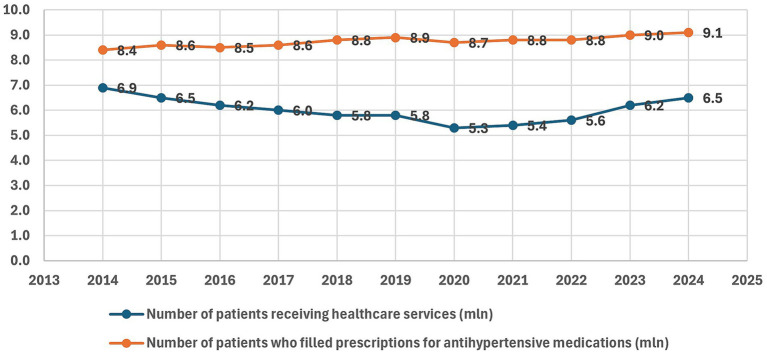
Number of adult patients (millions) receiving healthcare services with a primary diagnosis of dyslipidemia and number of adults who filled prescriptions for reimbursed lipid-lowering medications, 2014–2024, based on data from the National Health Fund (NHF) (37). X-axis: year; Y-axis: number of patients (millions).

Obesity is closely linked with dyslipidemia, with large epidemiologic studies indicating substantially higher odds of lipid abnormalities among individuals with excess body weight (42, 43). Figure 7 shows the number of adult patients in Poland with a primary diagnosis of dyslipidemia who received healthcare services, compared with the number who filled prescriptions for reimbursed lipid-lowering medications between 2014 and 2024. Prescription uptake followed a steady upward trajectory, rising from 4.6 million in 2014 to 6.5 million in 2024. In contrast, the number of patients receiving healthcare services fluctuated around 1.0–1.1 million for most of the period, with a marked decline in 2020 (748 thousand), coinciding with the COVID-19 pandemic, followed by a rebound to 1.26 million in 2024. Overall, these data indicate a continuous rise in the use of lipid-lowering medications alongside relatively stable, but recently increasing, healthcare service utilization for dyslipidemia.

In segmented log-linear time-series analysis, the pre-tax period showed a modest downward trend of approximately 1.8% per year in the number of patients receiving dyslipidemia-related services (β_time = −0.0185; 95% CI − 0.0375 to 0.0003; *p* = 0.053). After accounting for this pattern, the sugar-tax year 2021 was not associated with a statistically significant immediate change in level (β_post = 0.051; 95% CI − 0.061 to 0.164; *p* = 0.31). However, the slope became substantially steeper after 2021, with an additional annual increase of about 11.2% (β_slope post = 0.106; 95% CI 0.066 to 0.147; p ≈ 0.0006), consistent with a shift from a slightly declining to a clearly rising trend. The COVID-19 year 2020 was associated with an estimated 23% reduction in the number of patients compared with the underlying trend (β_covid2020 = −0.259; 95% CI − 0.367 to −0.151; p ≈ 0.0011). As with other outcomes, these changes likely reflect a combination of pandemic-related disruptions and broader system-level factors rather than a direct causal effect of the sugar tax. Full regression results are provided in Supplementary Table S6.

#### 3.4.4 Knee osteoarthritis requiring endoprosthesis

Obesity is a well-established risk factor for knee osteoarthritis and for the need for joint replacement, with studies showing that higher body weight is associated with a greater risk of developing symptomatic knee disease (44).

**Figure 8:**
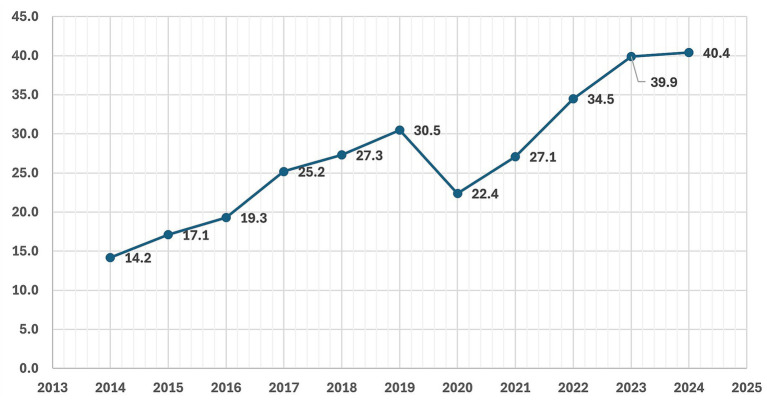
Number of adult patients (thousands) who underwent knee arthroplasty due to knee osteoarthritis in Poland between 2014 and 2024, based on data from the National Health Fund (NHF) (37). X-axis: year; Y-axis: number of patients (thousands).

According to NHF data (37) (Figure 8), the number of adult patients in Poland who underwent knee arthroplasty due to knee osteoarthritis rose substantially between 2014 and 2024, from 14.2 thousand to 40.4 thousand. A temporary decline was observed in 2020 (22.4 thousand), coinciding with the COVID-19 pandemic, but the trend subsequently recovered and accelerated, reaching its highest value in 2023 and remaining high in 2024. Overall, these data indicate a marked long-term increase in the surgical treatment of knee osteoarthritis.

In segmented log-linear time-series analysis (Supplementary Table S7), knee arthroplasty rates showed a strong pre-2020 upward trend, a pronounced pandemic-related drop in 2020–2021, and a subsequent return to the previous growth trajectory, without evidence of an additional structural break in level or slope after 2021. These patterns are most consistent with pandemic-related disruptions and recovery of elective surgery capacity rather than a direct effect of the sugar tax on arthroplasty rates.

Hospitals in Poland have recently been required to report BMI values for patients undergoing knee arthroplasty. The most recent NHF data (37) show that the mean BMI of women undergoing this procedure was 31.2 (median 30.9), while in men it was 30.8 (median 30.4). These findings clearly confirm that overweight and obesity are highly prevalent among patients treated surgically for knee osteoarthritis in Poland.

#### 3.4.5 Sleep apnea

**Figure 9:**
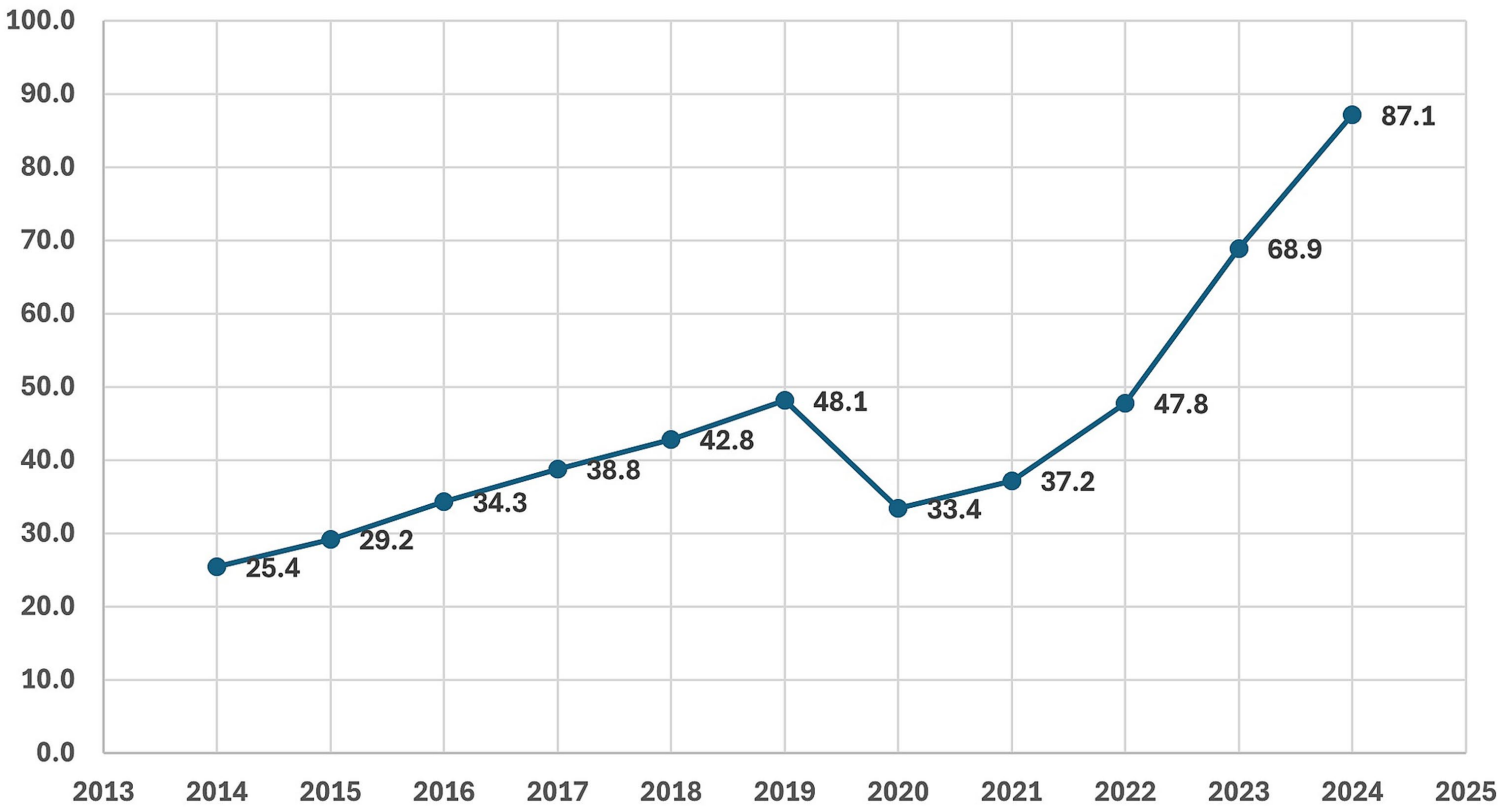
Number of adult patients (thousands) receiving healthcare services due to sleep apnea in Poland between 2014 and 2024, based on data from the National Health Fund (NHF) (37). X-axis: year; Y-axis: number of patients (thousands).

There is a well-established relationship between sleep apnea and excess body weight. Both overweight and obesity substantially increase the risk of obstructive sleep apnea (OSA), with strong evidence of a dose–response gradient between body mass index (BMI) and disease severity. Weight gain has also been identified as a significant predictor of OSA incidence and progression (45, 46). According to NHF data (37) (Figure 9), the number of adult patients in Poland receiving healthcare services due to sleep apnea increased markedly between 2014 and 2024. The figures rose from 25.4 thousand in 2014 to 48.1 thousand in 2019, before a temporary decline in 2020 (33.4 thousand), coinciding with the COVID-19 pandemic. From 2021 onwards, the upward trend resumed and accelerated, reaching 87.1 thousand in 2024 — the highest value recorded during the study period. Overall, these data point to a substantial and accelerating rise in the diagnosis and treatment of sleep apnea in Poland.

Segmented log-linear time-series analysis confirmed a steep pre-2020 increase in sleep apnea–related service use, a marked pandemic-related decline in 2020, and an accelerated post-2021 rise, consistent with expanding diagnosis and treatment activity after the COVID-19 disruption rather than a discrete effect attributable to the sugar tax. Full regression results are provided in Supplementary Table S8.

#### 3.4.6 Metabolic dysfunction-associated steatotic liver disease (MASLD)

**Figure 10:**
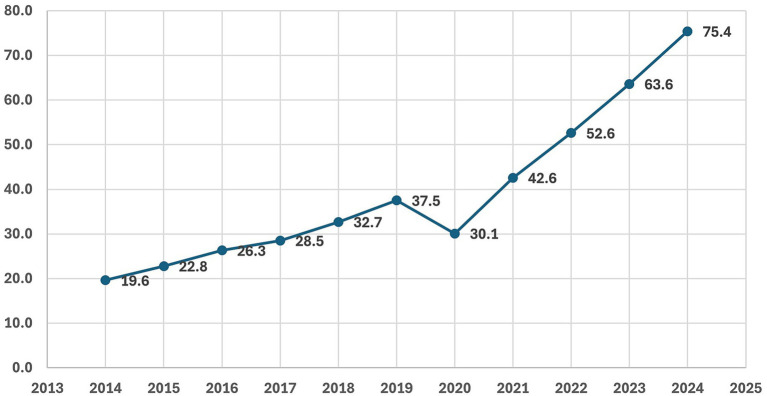
Number of adult patients (thousands) receiving healthcare services due to MASLD in Poland between 2014 and 2024, based on data from the National Health Fund (NHF) (37). X-axis: year; Y-axis: number of patients (thousands).

Scientific evidence indicates a strong association between obesity and MASLD. A recent global meta-analysis estimated that approximately 58% of adults with obesity are affected by MASLD (95% CI: 43.6–70.9%) (47). According to NHF data (37) (Figure 10), the number of adult patients in Poland receiving healthcare services due to MASLD increased markedly between 2014 and 2024. The values rose from 19.6 thousand in 2014 to 37.5 thousand in 2019, followed by a temporary decline in 2020 (30.1 thousand), coinciding with the COVID-19 pandemic. From 2021 onwards, the trend reversed and accelerated, reaching 75.4 thousand in 2024 — the highest level observed during the study period. Overall, these findings indicate a strong and accelerating upward trend in MASLD-related healthcare utilization in Poland.

Segmented time-series analysis (Supplementary Table S9) showed a strong pre-2020 upward trend in MASLD-related service use, a clear dip in 2020–2021, and a considerably steeper post-2021 increase, most plausibly reflecting growing recognition and coding of MASLD and recovery from pandemic-related disruptions, rather than a direct causal effect of the sugar tax.

## 4 Discussion

As summarized in Section 3.1, external market monitoring data indicate that the introduction of the sugar tax in Poland was followed by higher retail prices and modest reductions in sales volumes of sweetened non-alcoholic beverages, while NPHI survey data showed an initial decrease and subsequent partial rebound in self-reported daily SSB consumption (24, 27–29, 33). Taken together, these findings suggest some short-term responsiveness of consumption to fiscal and price signals, but also highlight that longer-term patterns are shaped by a broader mix of factors, including inflation and evolving consumer preferences, rather than by the tax alone.

The interpretation of these findings should also take into account the expected temporal dynamics of policy effects. Sugar-sweetened beverage taxes are likely to influence health outcomes through intermediate mechanisms, including changes in prices, purchasing behavior, and potentially product composition. While such proximal effects may occur relatively soon after implementation, downstream outcomes such as obesity prevalence and related chronic diseases are likely to evolve more gradually at the population level. In this context, the absence of clear short-term changes in obesity trends in our study should be interpreted with caution. For this reason, our findings should be regarded as hypothesis-generating rather than as evidence of causal policy effects.

The interpretation of these findings should also take into account the expected temporal dynamics of policy effects. Sugar-sweetened beverage taxes are likely to influence health outcomes through intermediate mechanisms, including changes in prices, purchasing behavior, and potentially product composition (19) While such proximal effects may occur relatively soon after implementation, as suggested by evidence on product reformulation following SSB tax policies in Europe (15), downstream outcomes such as obesity prevalence and related chronic diseases are likely to evolve more gradually at the population level. In this context, the absence of clear short-term changes in obesity trends in our study should be interpreted with caution.

In 2021–2023, the sugar tax (sugar levy) funds allocated to the NHF budget amounted to PLN 4.47 billion (24, 33). However, the NHF did not establish a separate system for monitoring, recording, and accounting for funds from taxes on sugar-sweetened beverages (SSBs) and alcoholic beverages (both included in the same report). These funds were only identifiable on the revenue side; on the expenditure side, they remained unallocated, thus entering a common pool. As a result, it was impossible to determine how much was allocated and for what specific health care purpose. The only identified expenditure from the sugar levy was for the implementation of the so-called KOS-BAR pilot program (coordinated care for surgical treatment of patients with morbid obesity), amounting to PLN 101.9 million (33).

Lack of clear allocation becomes particularly problematic considering the growing prevalence of obesity and related comorbidities, which significantly increase the demand for healthcare services. Rising rates of obesity contribute to a higher burden on the healthcare system, with associated costs for treating diseases such as diabetes, cardiovascular diseases, and other chronic conditions. The funds from the sugar tax could have been more effectively allocated to address these growing challenges, particularly through preventive measures and increased healthcare resources targeting obesity-related comorbidities. The funds from the sugar tax in Poland were not an additional source of financing (33), as they were intended to be spent (20), apart from health insurance contributions, for the prevention and treatment of diseases resulting from unhealthy behavior. That means, that the introduction of new mechanisms for raising funds did not contribute to a real increase in expenditure on the purpose specified in the act introducing levies on sweetened beverages (24, 33). These fiscal and governance aspects are discussed as policy context and do not imply a direct causal relationship with short-term changes in SSB consumption or healthcare service use in our time-series data. Therefore, there is no justification for any conclusions about the impact of the sugar tax on improving the health of patients with obesity and related chronic diseases in Poland. The increase in healthcare demand, as presented in this paper, and expected costs for the treatment of chronic diseases related to obesity, does not mean that the sugar tax has proved ineffective. The sugar levy program is planned to be continued for many years, but its intended effectiveness will only be achievable if the funds raised are directed for the prevention and treatment of diseases resulting from unhealthy behavior and leading to obesity. It should also be considered that although bariatric surgery is an effective method of treating obesity, it is very costly and only benefits a small group of patients. It is also worth noting that the consumers of SSBs are often young people without chronic metabolic diseases (so far), thus a reduction in the consumption of these beverages will not translate into a decrease in the incidence of chronic diseases in the population within just a few years. Attention should be focused on using the funds from the sugar tax to prevent overweight, obesity and related disorders, and, on the other hand, for developing programs enabling the most seriously affected patients to benefit from reimbursed modern pharmacotherapy for obesity, which, as the SELECT study has shown for semaglutide, can significantly reduce the risk of cardiovascular events and mortality in patients with diagnosed cardiovascular disease (48).

This study has several important limitations that constrain the interpretation of the findings. In particular, the ecological design precludes inference at the individual level and is subject to ecological fallacy. The relatively short post-intervention period (4 years) limits the ability to detect longer-term effects. In addition, multiple concurrent factors, including the COVID-19 pandemic and changes in healthcare organization, may have influenced the observed trends.

As a further limitation, the observed increase in the number of patients receiving pharmacological treatment for obesity may only partly reflect the recent introduction and growing availability of modern anti-obesity medications in Poland. In 2019, several of these therapies were still relatively new and rarely used, which likely contributes to the sharp rise in prescription fulfilment observed from 2021 onwards, even before the introduction of semaglutide and tirzepatide for obesity treatment. Additionally, the COVID-19 pandemic and the increased risk of severe disease in patients with obesity may have raised awareness and prompted people to seek medical help for weight loss, potentially influencing the number of prescriptions fulfilled. It should be noted though that semaglutide and tirzepatide were not yet available for the treatment of obesity during the study period.

Furthermore, the data on bariatric surgery reflect only procedures reimbursed by the National Health Fund (NHF); surgeries performed in the private sector are not captured, which likely results in a systematic underestimation of the total number of procedures performed during the study period. It is also important to consider that the increase in the number of healthcare services related to overweight and obesity, including bariatric surgeries, occurred in parallel with increased financial allocations to the NHF. The observed growth indirectly reflects both the rising population demand for these services and the improved financial capacity of the public payer, supported by the revenues from the sugar tax. Additionally, from July 2021, the NHF abolished the limits on outpatient services, which likely facilitated greater access to obesity-related healthcare services, including consultations, diagnostic tests, and treatments. Limits, on the one hand, served as an effective tool to protect the payer from excessive financial burdens, but on the other hand, they restricted access to services (49), potentially limiting the supply of care for patients with obesity. The removal of these limits improved access to care but also presents a limitation in assessing the specific effects of the sugar tax, as it complicates the interpretation of healthcare utilization patterns. The increased healthcare demand following the removal of limits highlights the ongoing challenge of obesity, which aligns with our findings.

Finally, the aging population may also play a role in the observed trends, as older individuals are more likely to suffer from obesity and related comorbidities, further increasing the demand for healthcare services.

It should also be emphasized that the introduction of the sugar tax in 2021 and the availability of data only through 2024 represent a relatively short observation period, with only four post-tax years currently observable. In addition, all analyses are based on nationally aggregated data from separate administrative and survey sources, without individual-level linkage. As a result, we cannot determine whether individuals who reduced or increased SSB consumption are the same individuals who developed obesity or obesity-related comorbidities, whether changes in SSB consumption occurred in the same demographic subgroups that experienced changes in obesity prevalence, or whether patients with type 2 diabetes, hypertension, dyslipidemia, sleep apnea, knee osteoarthritis or MASLD have ever been regular SSB consumers. Our findings therefore reflect population-level temporal associations and should not be interpreted as individual-level causal relationships. Moreover, the years 2020 and 2021 were strongly affected by the COVID-19 pandemic, which disrupted both healthcare utilization and reporting systems, making interpretation of trends in this period more difficult. Finally, the NHF data are administrative in nature and subject to potential coding inconsistencies, and some indicators used in this study represent proxy measures (e.g., prescription fulfillment as a proxy for treatment, per-capita sugar consumption as a proxy for individual intake), which may not fully capture the underlying epidemiological reality. In addition, we did not have access to detailed, product- or category-specific data on SSB prices and purchasing patterns, or to information broken down by demographic groups. We also did not have access to product-level data on sugar content, which prevented assessment of potential reformulation effects. To our knowledge, such fine-grained price and purchasing data are not available in the open public domain and, if available, would most likely be contained in commercial market research datasets and industry reports. This lack of granular price and purchasing information represents an additional limitation of our analysis and prevented us from performing detailed CPI–SSB price comparisons or modelling behavioral responses by subgroup. The limited number of observation points for this analysis also highlights the need for more extended monitoring, ideally covering several years before and after the introduction of the sugar tax. Unfortunately, such data are currently lacking, making it difficult to fully assess the long-term effects of this fiscal intervention. Taken together, these limitations highlight the need for large-scale, population-based public health studies in Poland to more precisely assess the long-term effects of fiscal interventions such as the sugar tax on obesity, dietary behaviors, and associated health outcomes.

Future comparative analyses across European countries may help distinguish country-specific patterns from broader behavioral responses to sugar taxation policies.

## 5 Conclusion

This study highlights that, over the first 4 years following the introduction of the sugar tax in Poland (2021–2024), the prevalence of overweight and obesity has continued to rise, accompanied by a growing burden of obesity-related comorbidities such as type 2 diabetes, hypertension, dyslipidemia, knee osteoarthritis, sleep apnea, and MASLD. While sugar-sweetened beverage consumption initially declined and per-capita sugar purchases decreased modestly over 2018–2024, our analyses did not show a distinct structural break in this series after 2021. Persistently high consumption of sweets and other processed foods may have limited the extent of reductions in total sugar exposure, but this potential compensation effect cannot be quantified with the ecological data available. At the same time, healthcare utilization for obesity management increased, reflected in rising numbers of bariatric procedures and expanding pharmacological treatment, yet these efforts have clearly not been sufficient to halt the upward trends in overweight and obesity. Most effective modern anti-obesity medications, such as semaglutide and tirzepatide, were not available for obesity treatment or reimbursed during the study period and therefore could not materially influence the observed population-level trends. Within the constraints of this ecological time-series design and the relatively short post-tax follow-up, these findings should be interpreted as descriptive, population-level temporal associations in the context of sugar tax implementation, rather than as evidence of either effectiveness or ineffectiveness of the tax at the individual level. Even if a direct cause-and-effect relationship cannot be proven, fiscal measures that reduce the consumption of sweetened beverages are likely to be beneficial for population health when embedded in broader strategies.

The experience to date in Poland suggests that a sugar tax alone is unlikely to curb the obesity epidemic; its potential depends on sustained implementation, transparent allocation of revenues to prevention and care, and integration with wider public health actions, including health education, promotion of healthy dietary habits, and adequate access to effective obesity treatment. Comprehensive approaches that combine fiscal policies with clinical, preventive, and educational measures are essential to address the rising prevalence of obesity and its associated health consequences in Poland.

## Data Availability

The original contributions presented in the study are included in the article/Supplementary material, further inquiries can be directed to the corresponding author.
