# Supplementary material for: Sugar tax in Poland: population-level trends in obesity, sugar consumption and healthcare utilization
Source: Front Public Health. 2026 Apr 28;14:1811509. doi: 10.3389/fpubh.2026.1811509 (PMC13160712; doi:10.3389/fpubh.2026.1811509)
Supplement: Supplementary file 1 [file Supplementary_file_1.docx]

# **Supplementary Material**

**Table S0. Summary of indicators, data sources, aggregation level and analysis type**

| **Indicator** | **Data source** | **Variable definition / unit** | **Aggregation level** | **Years available** | **Type of data** | **Analysis in this study** |
| --- | --- | --- | --- | --- | --- | --- |
| Per-capita sugar purchases | CSO | Annual per-capita sugar consumption (kg per person per year) | National population, annual per-capita estimate | 2018–2024 | Aggregated | Segmented time-series regression; descriptive trends |
| SSB consumption frequency (overall, by sex) | NPHI | Weighted % of adults reporting given frequency of SSB consumption (Everyday, ≥1x/week, <1x/week, none) | National survey, aggregated weighted proportions | 2018, 2022, 2025 | Aggregated | Descriptive statistics; no ITS |
| Sweets consumption frequency (overall, by sex) | NPHI | Weighted % of adults reporting given frequency of sweets consumption | National survey, aggregated weighted proportions | 2018, 2022, 2025 | Aggregated | Descriptive statistics; no ITS |
| Overweight and obesity prevalence | NPHI | Weighted % of adults with BMI ≥25 kg/m² (overweight) and ≥30 kg/m² (obesity) | National survey, aggregated weighted proportions | 2018, 2022, 2025 | Aggregated | Descriptive statistics; no ITS |
| BMI distribution (general adult population, 2025) | NPHI | Cross-sectional distribution of BMI categories in 2025 | National survey, aggregated distribution | 2025 (7 months) | Aggregated | Descriptive statistics; no ITS |
| Patients receiving bariatric surgery | NHF | Annual number of unique adult patients with reimbursed bariatric procedures | National population, annual count of unique patients | 2014–2024 | Aggregated | Segmented time-series regression |
| Patients receiving obesity pharmacotherapy | e-Health Center | Annual number of unique patients filling prescriptions for reimbursed obesity medications | National population, annual count of unique patients | 2019–2024 | Aggregated | Simplified segmented time-series regression |
| Patients with T2D – healthcare services | NHF | Annual number of unique adult patients receiving T2D-related healthcare services | National population, annual count of unique patients | 2014–2024 | Aggregated | Segmented time-series regression |
| Patients with T2D – medications / test strips | NHF | Annual number of unique adult patients purchasing diabetes medications or glucose test strips | National population, annual count of unique patients | 2014–2024 | Aggregated | Descriptive trends; contextual to service use |
| Patients with hypertension – healthcare services | NHF | Annual number of unique adult patients with primary diagnosis of hypertension receiving healthcare services | National population, annual count of unique patients | 2014–2024 | Aggregated | Segmented time-series regression |
| Patients with dyslipidemia – healthcare services | NHF | Annual number of unique adult patients with primary diagnosis of dyslipidemia receiving healthcare services | National population, annual count of unique patients | 2014–2024 | Aggregated | Segmented time-series regression |
| Patients with sleep apnea – healthcare services | NHF | Annual number of unique adult patients receiving healthcare services due to sleep apnea | National population, annual count of unique patients | 2014–2024 | Aggregated | Segmented time-series regression |
| Knee arthroplasty due to osteoarthritis | NHF | Annual number of unique adult patients undergoing knee arthroplasty with knee osteoarthritis as indication | National population, annual count of unique patients | 2014–2024 | Aggregated | Segmented time-series regression |
| MASLD-related healthcare services | NHF | Annual number of unique adult patients receiving healthcare services due to MASLD | National population, annual count of unique patients | 2014–2024 | Aggregated | Segmented time-series regression |
| BMI among knee arthroplasty patients | NHF | Mean, median and distribution of BMI among patients undergoing knee arthroplasty | National, aggregated BMI summary for operated patients | Most recent year(s) available | Aggregated | Descriptive statistics; no ITS |

**Table S1. Segmented time-series regression results for per-capita sugar consumption in Poland, 2018–2024.**

| **Parameter** | **Interpretation** | **Coefficient  β (log scale)** | **Standard  error** | **95% CI  for β** | **p-value** | **Approx. % change** |
| --- | --- | --- | --- | --- | --- | --- |
| Intercept | ln(kg per capita) in 2018 | 3.85 | 0.04 | 3.68 to 4.02 | <0.001 | - |
| Time | Pre-tax annual time trend (2018–2020) | −0.11 | 0.06 | −0.35 to 0.13 | 0.19 | −10.4% |
| Post2021 | Immediate level change in 2021 vs. expected value | 0.24 | 0.15 | −0.39 to 0.86 | 0.25 | +26.6% |
| Time_after2021 | Change in slope after 2021 (additional annual trend) | 0.10 | 0.06 | −0.15 to 0.35 | 0.24 | +10.1% |
| Covid2020 | Deviation in 2020 from the underlying pre-tax trend | 0.13 | 0.10 | −0.28 to 0.54 | 0.31 | +13.8% |

**Table S2. Segmented time-series regression for the number of reimbursed bariatric procedures, Poland 2017–2024**

| **Parameter** | **Interpretation** | **Coefficient  β (log scale)** | **Standard  error** | **95% CI  for β** | **p-value** | **Approx. % change** |
| --- | --- | --- | --- | --- | --- | --- |
| Intercept | ln((number of procedures) in 2017 | 8.10 | 0.027 | 8.01 to 8.18 | <0.001 | - |
| Time | Pre-tax annual trend (per year) | 0.1551 | 0.0206 | 0.0895 to 0.2207 | 0.0049 | +16.8% |
| Post2021 | Immediate level change in 2021 vs. expected value | -0.1875 | 0.0686 | −0.4057 to 0.0308 | 0.0717 | -17.1% |
| Time_after2021 | Change in slope after 2021 (additional annual trend) | -0.0224 | 0.0244 | −0.1000 to 0.0552 | 0.4261 | -2.2% |
| Covid2020 | Deviation in 2020 from the underlying pre-tax trend | -0.3736 | 0.0532 | −0.5430 to −0.2041 | 0.0059 | -31.2% |

**Table S3. Segmented time-series regression for the number of patients receiving reimbursed pharmacotherapy for obesity, Poland 2019–2024**

| **Parameter** | **Interpretation** | **Coefficient β  (log scale)** | **Standard  error** | **95% CI  for β** | **p-value** | **Approx. % change** |
| --- | --- | --- | --- | --- | --- | --- |
| Intercept | ln(number of patients) in 2019 | 3.10 | 0.30 | 1.80 to 4.39 | 0.0093 | - |
| Time | Annual time trend (2019–2024) | 0.32 | 0.13 | -0.26 to 0.90 | 0.14 | +37.8% |
| Post2021 | Level difference for 2021–2024 vs. 2019–2020 | 0.40 | 0.58 | -2.08 to 2.89 | 0.56 | +49.8% |
| Covid2020 | Deviation in 2020 from the underlying trend | -0.21 | 0.45 | -2.13 to 1.71 | 0.68 | -18.9% |

**Table S4. Segmented time-series regression for the number of adult patients receiving diabetes-related healthcare services, Poland 2014–2024**

| **Parameter** | **Interpretation** | **Coefficient β  (log scale)** | **Standard  error** | **95% CI  for β** | **p-value** | **Approx. % change** |
| --- | --- | --- | --- | --- | --- | --- |
| Intercept | ln(number of patients) in 2014 | 0.565 | 0.010 | 0.539 to 0.591 | 2.7×10⁻⁹ | - |
| Time | Pre-tax annual trend (2014–2020) | 0.0054 | 0.0035 | -0.0031 to 0.0139 | 0.17 | +0.5% |
| Post2021 | Immediate level change in 2021 vs. expected value | -0.0341 | 0.0206 | -0.0845 to 0.0164 | 0.15 | −3.3% |
| Time_after2021 | Change in slope after 2021 (additional annual trend) | 0.0507 | 0.0073 | 0.0327 to 0.0687 | 0.00046 | +5.2% |
| Covid2020 | Deviation in 2020 from the underlying pre-tax trend | -0.0667 | 0.0198 | -0.1151 to -0.0183 | 0.015 | −6.5% |

**Table S5. Segmented time-series regression for the number of adult patients receiving hypertension-related healthcare services, Poland 2014–2024**

| **Parameter** | **Interpretation** | **Coefficient β  (log scale)** | **Standard  error** | **95% CI  for β** | **p-value** | **Approx. % change** |
| --- | --- | --- | --- | --- | --- | --- |
| Intercept | ln(number of patients) in 2014 | 1.91 | 0.014 | 1.88 to 1.95 | 1.1×10⁻¹¹ | - |
| Time | Pre-tax annual trend (2014–2020) | -0.036 | 0.0047 | -0.047 to -0.024 | <0.001 | −3.5% |
| Post2021 | Immediate level change in 2021 vs. expected value | 0.014 | 0.0279 | -0.053 to 0.083 | 0.61 | +1.4% |
| Time_after2021 | Change in slope after 2021 (additional annual trend) | 0.101 | 0.0099 | 0.077 to 0.126 | <0.001 | +10.7% |
| Covid2020 | Deviation in 2020 from the underlying pre-tax trend | -0.031 | 0.0268 | -0.096 to 0.035 | 0.30 | −3.0% |

**Table S6. Segmented time-series regression for the number of adult patients receiving dyslipidemia-related healthcare services, Poland 2014–2024**

| **Parameter** | **Interpretation** | **Coefficient β  (log scale)** | **Standard  error** | **95% CI  for β** | **p-value** | **Approx. % change** |
| --- | --- | --- | --- | --- | --- | --- |
| Intercept | ln(number of patients) in 2014 | 6.98 | 0.023 | 6.93 to 7.05 | 9.5×10⁻¹⁴ | - |
| Time | Pre-tax annual trend (2014–2020) | -0.0185 | 0.0077 | -0.0375 to 0.0003 | 0.053 | −1.8% |
| Post2021 | Immediate level change in 2021 vs. expected value | 0.0513 | 0.0460 | -0.0612 to 0.1638 | 0.31 | +5.3% |
| Time_after2021 | Change in slope after 2021 (additional annual trend) | 0.1064 | 0.0164 | 0.0664 to 0.1465 | 0.00063 | +11.2% |
| Covid2020 | Deviation in 2020 from the underlying pre-tax trend | -0.259 | 0.0442 | -0.367 to -0.151 | 0.0011 | −22.8% |

**Table S7. Segmented time-series regression for the number of adult patients undergoing knee arthroplasty due to knee osteoarthritis, Poland 2014–2024**

| **Parameter** | **Interpretation** | **Coefficient β  (log scale)** | **Standard  error** | **95% CI  for β** | **p-value** | **Approx. % change** |
| --- | --- | --- | --- | --- | --- | --- |
| Intercept | ln(number of patients) in 2014 | 2.67 | 0.045 | 2.57 to 2.78 | <0.001 | - |
| Time | Pre-tax annual trend (2014–2020) | 0.16 | 0.015 | 0.121 to 0.193 | 4.1×10⁻⁵ | +17.0% |
| Post2021 | Immediate level change in 2021 vs. expected value | -0.42 | 0.088 | -0.633 to -0.204 | 0.0031 | −34.2% |
| Time_after2021 | Change in slope after 2021 (additional annual trend) | -0.022 | 0.031 | -0.099 to 0.054 | 0.50 | −2.2% |
| Covid2020 | Deviation in 2020 from the underlying pre-tax trend | -0.51 | 0.084 | -0.714 to -0.301 | 0.00096 | −39.8% |

**Table S8. Segmented time-series regression for the number of adult patients receiving healthcare services due to sleep apnea, Poland 2014–2024**

| **Parameter** | **Interpretation** | **Coefficient β  (log scale)** | **Standard  error** | **95% CI  for β** | **p-value** | **Approx. % change** |
| --- | --- | --- | --- | --- | --- | --- |
| Intercept | ln(number of patients) in 2014 | 3.25 | 0.021 | 3.20 to 3.30 | <0.001 | - |
| Time | Pre-tax annual trend (2014–2020) | 0.11 | 0.007 | 0.097 to 0.130 | <0.001 | +12.0% |
| Post2021 | Immediate level change in 2021 vs. expected value | -0.54 | 0.041 | -0.64 to -0.44 | <0.001 | −41.7% |
| Time_after2021 | Change in slope after 2021 (additional annual trend) | 0.16 | 0.015 | 0.13 to 0.20 | <0.001 | +17.9% |
| Covid2020 | Deviation in 2020 from the underlying pre-tax trend | -0.51 | 0.040 | -0.61 to -0.41 | <0.001 | −40.0% |

**Table S9. Segmented time-series regression for the number of adult patients receiving healthcare services due to MASLD, Poland 2014–2024**

| **Parameter** | **Interpretation** | **Coefficient β  (log scale)** | **Standard  error** | **95% CI  for β** | **p-value** | **Approx. % change** |
| --- | --- | --- | --- | --- | --- | --- |
| Intercept | ln(number of patients) in 2014 | 2.99 | 0.013 | 2.96 to 3.02 | <0.001 | - |
| Time | Pre-tax annual trend (2014–2020) | 0.126 | 0.0043 | 0.116 to 0.136 | <0.001 | +13.4% |
| Post2021 | Immediate level change in 2021 vs. expected value | -0.110 | 0.0252 | -0.172 to -0.048 | 0.0048 | −10.4% |
| Time_after2021 | Change in slope after 2021 (additional annual trend) | 0.065 | 0.0090 | 0.042 to 0.086 | 0.00004 | +6.7% |
| Covid2020 | Deviation in 2020 from the underlying pre-tax trend | -0.342 | 0.0242 | -0.401 to -0.282 | 0.000008 | −28.9% |
